# Effects of Hydroxy-Alpha-Sanshool on Intestinal Metabolism in Insulin-Resistant Mice

**DOI:** 10.3390/foods11142040

**Published:** 2022-07-10

**Authors:** Fangyan Xu, Yuping Zhu, Mintao Lu, Likang Qin, Degang Zhao, Tingyuan Ren

**Affiliations:** 1 College of Brewing and Food Engineering, Guizhou University, Guiyang 550025, China; xufy980502@163.com (F.X.); lumintao0606@163.com (M.L.); lkqin@gzu.edu.cn (L.Q.); dgzhao@gzu.edu.cn (D.Z.); 2 School of Basic Medicine, Guizhou Medical University, Guiyang 550025, China; zhuyupingzhuyu@163.com; 3 Guiyang Station for DUS Testing Center of New Plant Varieties of the Ministry of Agriculture and Rural Affairs of the People’s Republic of China in Guizhou Academy of Agricultural Sciences, Guiyang 550006, China

**Keywords:** hydroxy-alpha-sanshool, insulin resistant, metabolism, intestines

## Abstract

To explore the hydroxy-alpha-sanshool (HAS) effects on the intestinal metabolites of insulin-resistant mice, the blank group (BG), model group (MG), and HAS dose group (DG) were designed. The insulin resistance (IR) model was induced through streptozotocin (STZ) combined with a high-fat and high-sugar diet. Based on the availability of the model, the HAS dose was given by gavage for 28 days. The determination of cecum and key serum indexes was made, including the contents of insulin (INS), triglycerides (TG), total cholesterol (TC), glycosylated serum protein (GSP), and glycosylated hemoglobin (GHb). The changes in gut microbiota and metabolites in cecal contents were detected by 16S rRNA gene amplicon sequencing and UPLC/HRMS technology, respectively. The results that the levels of GSP, GHb, TG, and TC were significantly increased; this was not the case for INS; or for the changes in the gut microbiota and metabolites in MG. However, the intervention of HAS effectively reversed these changes, for instance, it decreased levels of GSP, GHb, TG, TC, and alterations of metabolite composition for linoleic acid and tyrosine metabolism and recovered trends of declining species diversity and richness of the gut microbiota in MG. It was indicated that HAS alleviated IR by regulating the gut microbiota and metabolites and affecting lipid and amino acid metabolism pathways.

## 1. Introductions

In recent years, with societal development, people’s diets and lifestyles have undergone tremendous changes. The prolonged intake of high-sugar and high-fat diets and reduced physical activity have led to diabetes becoming a significant global health problem. Insulin resistance (IR) is an insulin-mediated glucose metabolism defect occurring in tissues. It is one of the earliest manifestations of several illnesses, including type 2 diabetes mellitus (T2DM) and cardiovascular disorders [1].

Several studies have shown that IR is a central link in the development of T2DM and the onset of the disease [2,3,4]. The IR is a pathological state closely related to obesity [5]. Therefore, interfering with obesity can improve the IR symptoms and effectively prevent the occurrence and development of T2DM. There is a close correlation between intestinal microbiome imbalance and IR during obesity. Studies have identified that intestinal immune homeostasis disorder caused by intestinal microbiota and mucosal dysfunction due to inflammation is closely correlated with the process of IR [6,7]. The intestine is vital for the digestion and extraction of nutrients such as lipids, carbohydrates, and proteins. However, its role in metabolic diseases has not been explored in detail. In addition, the intestinal microbiota of diabetic patients often has a structural disorder, which changes the metabolites. The intestinal microbiota and metabolites stimulate the body to produce cytokines, affecting glucose metabolism and insulin sensitivity in the body [8,9]. Therefore, improving intestinal microecological disorders can prevent and treat diabetes.

*Zanthoxylumbungeanum* Maxim is a fruit of *Zanthoxylum* genus from the Rutaceae family with a long history in China where it is used as cooking seasoning or medicine [10,11]. As a chain unsaturated fatty acid amide, hydroxyl-α-sanshool (HAS) was extracted from the peel of *Z. bungeanum* Maxim, its numbness was mainly attributable to HAS [11,12]. Studies have revealed that HAS can relax the annular muscles of the gastric body, contract the intestinal smooth muscle cells, promote small intestinal peristalsis, enhance intestinal motility and blood flow, and prevent adhesions after intestinal obstruction [13,14]. Furthermore, HAS also improves intestinal microbiota disorder caused by diabetes. Moreover, after intragastric administration of HAS, some probiotics in cecal contents significantly increased, while the population of pathogenic bacteria under certain conditions significantly decreased [15]. At present, the effect and mechanism of *Z. bungeanum* and HAS on bodily glucose [16], lipid [12], and protein [17] metabolism have been evident. However, only a few studies have been conducted on the effects of HAS on intestinal health. Therefore, studying the impact of the HAS on intestinal health is of great significance.

The present study explored the effect of HAS on regulating intestinal metabolic disorders in IR mice induced by streptozotocin (STZ) combined with a high-fat and high-sugar diet. In addition, 16S rRNA gene amplicon sequencing and metabonomics explore the effect and mechanism of HAS in the adjuvant treatment of insulin resistance and provide a scientific basis for the development and utilization of HAS.

## 2. Material and Methods

### 2.1. Animals

Thirty-five healthy 4–6 weeks old male Kunming mice with a bodyweight of 20–22 g were provided by Chongqing Tengxin Biotechnology Co., Ltd. (Chongqing, China). During the adaption period, the animals were fed under controlled temperature (22 ± 2 °C), controlled humidity (45–65%), and a 12 h/12 h day-night cycle. Water and diet were provided ad libitum. The experiment was approved by the Animal Experiment Ethics Committee of Guizhou University (Approval No. EAE-GZU-2020-P003), conforming to animal experimentation ethics.

### 2.2. Materials

HAS was procured from Shanghai Yuanye Biotechnology Co., Ltd., Shanghai, China, CAS No. 83883-10-7 and article number B26430 (HPLC ≥ 98%). Streptozotocin (STZ) was obtained from Sigma-Aldrich (St. Louis, MO, USA).

### 2.3. Insulin Resistance Model

After one week of adaptive feeding with a basic diet, the mice were randomly divided into a blank group (*n* = 10) and a high-fat and high-sugar group (*n* = 25) based on the bodyweight of the mice. A high-fat and high-sugar diet united with STZ established the IR model. We prepared an STZ solution at a concentration of 6 mg/mL with citric acid buffer and the STZ was intraperitoneally injected at the weight of 1 mL/100 g of mice. After feeding the high-fat and high-sugar diet for eight weeks, the mice were fasted for 12 h. The blank group was intraperitoneally injected with citric acid buffer, and the high-fat and high-sugar group was intraperitoneally injected with 60 mg/kg·bw STZ. During modeling, water and diet were provided ad libitum. On the 7th day after modeling and fasting for 12 h, blood samples were collected from the tip of the tail, and fasting blood glucose (FBG) was measured with a glucometer. The modeling was successful when the FBG value was higher than 11.10 mmol/L [18], and the mice with failed modeling were screened. The 20 successfully modeled mice were divided into two groups, viz., the model group, and the HAS dose group (8 mg/kg·bw), with ten mice in each group. The weight of the mice was recorded every week. The preliminary studies of this project showed that the intragastric dose of 4~12 mg/kg·bw had an effect on lipids, blood glucose, and protein, among which 8 mg/kg·bw had a significant effect on lipid, sugar, and protein metabolism in mice [16,19,20]. Therefore, the intragastric dose in this study was based on the results of previous studies.

### 2.4. Mice Diets

The three experimental groups were categorized as the blank group (BG), the model group (MG), and the HAS dose group (DG). The laboratory HAS was intragastric at the weight of 1 mL/100 g of mice. The HAS standard was dissolved in edible soybean oil to prepare the solution with a concentration of 0.8 mg/mL. The DG was given 0.8 mg/mL HAS intragastric administration from 9:00 to 11:00 every morning. The BG and MG were provided the edible soybean oil through intragastric administration. The BG was supplied a basal diet, and the other groups were provided a high-fat and high-sugar diet. The formula of high-fat and high-sugar feed for mice was devised by referring to the “Procedure and Test Method of Functional Evaluation of Health Food”. Water and diet were provided ad libitum during the experiment, which lasted for 28 days.

### 2.5. Sample Collection

On the 29th day, the blood samples were collected from the eyeballs of mice fasting for 12 h and kept in a centrifuge tube. The samples were rapidly centrifuged within 0.5 h at 4000 r/min, at 4 °C for 15 min. The upper serum was stored at −80 °C for later detection. The serum consisted of triglycerides (TG), total cholesterol (TC), high-density lipoprotein cholesterol (HDL-C), glycosylated serum protein (GSP), low-density lipoprotein cholesterol (LDL-C), glycosylated hemoglobin (GHb), and insulin (INS) were determined using the ELISA kits (Jiancheng, Nanjing, China). The mice were sacrificed by cervical dislocation, the cecum was dissected, weighed, and cut lengthwise, then the cecal contents were placed in a sterilized EP tube, which was frozen with a liquid nitrogen box, and finally transferred to a −80 °C refrigerator for the determination of 16S rRNA gene amplicon sequencing and metabolomics of the cecal contents. Then immediately clean the cecal tissue with cold saline, dry the surface water with absorbent paper, weigh the cecal tissue wet weight, wrap it in tin foil paper and store it at −4 °C for the determination of the cecal surface area. The wall surface area of the cecum was measured with reference to You Ym et al. [15]. The small intestine was removed and stored in 10% formalin until processed in wax blocks. Then, the small intestine was stained with hematoxylin and eosin (HE) after sectioning to examine the villus structure.

### 2.6. 16S rRNA Gene Amplicon Sequencing 

#### 2.6.1. DNA Extraction

The total genomic DNA from cecal contents was extracted using the OMEGA Soil DNA Kit (M5635–02) (Omega Bio-Tek, Norcross, GA, USA), stored at −20 °C, and following the manufacturer’s instructions before further analysis. The quantity and quality of extracted DNAs were analyzed with a NanoDrop NC 2000 spectrophotometer (Thermo Fisher Scientific, Waltham, MA, USA) and agarose gel electrophoresis, respectively. 

#### 2.6.2. 16S rRNA Gene Amplicon Sequencing and Analysis

Mice cecal contents are collected during dissection, and during transport are preserved in dry ice after liquid nitrogen quick-freeze, and 16S rRNA gene amplicon sequencing of the intestinal microbiota is performed to analyze changes in the intestinal microbiota of mice. The sequencing steps were completed by Sichuan Panomik Biotechnology Co., Ltd. (Chengdu, China).

The V3–V4 region of the bacterial 16S rRNA genes was amplified through PCR using 338F (5’-ACTCCTACGGGAGGCAGCA-3’) and 806R (5’-GGACTACHVGGGTWTCTAAT-3’) as forward and reverse primers, respectively. PCR amplicons were purified using the Vazyme VAHTSTM DNA Clean Beads (Vazyme, Nanjing, China) and quantified with the Quant-iT PicoGreen dsDNA Assay Kit (Invitrogen, Carlsbad, CA, USA). After the individual quantification step, amplicons were pooled in equal amounts, and 2 × 250 bp pair-end sequencing was undergone using the Illumina MiSeq platform with MiSeq Reagent Kit v3 at the Shanghai Personal Biotechnology Co., Ltd. (Shanghai, China).

Microbiome bioinformatics were performed with QIIME2 2019.4 [21] with slight modifications according to the official tutorials. Briefly, raw sequence data were demultiplexed using the demux plugin followed by primers cutting with a cut adapt plugin. Sequences were then quality filtered, denoised, merged and chimera removed using the DADA2 plugin [22]. Sequence data were mainly analyzed using QIIME2 and R packages (v3.2.0). ASV-level alpha diversity indices, including chao1 richness estimator, observed-species, ACE, Shannon diversity index, and PD-whole-tree index, were determined using the ASV table in QIIME2 and visualized as bar graphs. The taxonomy compositions and abundances were determined and visualized using MEGAN [23] and GraPhlAn [24].

### 2.7. Metabonomics Analysis

#### 2.7.1. Metabolite Extraction and Mix Standard Curve Preparation

Exactly 100 mg (±1%) of the cecal contents were weighed accurately into a 2 mL eppendorf (EP) tube, followed by adding 0.6 mL of methanol (including internal standard) and 100 mg of glass beads, which were vortexed for 1 min (Qilinbeier, BE-2600). The solution was grounded at 50 Hz for 60 s in a high-throughput tissue grinder (MEIBI, MB-96) and repeated twice. Then, ultrasound was performed at room temperature for 15 min and centrifuged (Cence, H2050-R) at 4 °C, 12,000 rpm for 10 min. After that, 200 µL supernatant was taken and added to the detector bottle. Later, 20 µL was taken from each sample to the quality control (QC) samples (These QC samples monitored deviations of the analytical results from these pool mixtures and were compared to the errors of the analytical instrument).

The same amount of sample supernatant prepared by “metabolite extraction” was taken into a 2 mL centrifuge tube and vortexed for 1 min. Then, the mixed sample was diluted with methanol (including internal standard) as diluent based on 0.8 * cal 1, 0.5 * cal 1, 0.4 * cal 1, 0.2 * cal 1, and 0.1 * cal 1. Six standard curve points were provided and were arranged to be tested together.

#### 2.7.2. Chromatographic and Mass Spectrometry Conditions

Chromatographic separation was used with an ACQUITY UPLC^®^ HSS T3 (150 × 2.1 mm, 1.8 µm) (Waters, Milford, MA, USA) column temperature maintained at 40 °C. The temperature of the autosampler was 8 °C. Gradient elution of analytes was carried out with 0.1% formic acid in water © and 0.1% formic acid in acetonitrile (D) or 5 mM ammonium formate in water (A) and acetonitrile (B) at a flow rate of 0.25 mL/min. Injection of 2 µL of each sample was done after equilibration. An increasing linear gradient of solvent B (*v*/*v*) was used as follows: 0~1 min, 2% B/D, 98% A/C; 1~9 min, 2~50% B/D, 98~50%, A/C; 9~12 min, 50~98% B/D, 50~2% A/C; 12~13.5 min, 98% B/D, 2% A/C; 13.5~14 min, 98~2% B/D, 2~98%, A/C; and 14~20 min, 2% D, 98% C-positive model (14~17 min, 2% B, 98% A-negative model). (Thermo, U3000).

The ESI-MSn experiments were conducted in positive and negative ion scanning modes with a positive ion mode of 3.5 kV and a negative ion mode of −2.5 kV. Both sheath and auxiliary gases were set at 30 and 10 arbitrary units, respectively. The capillary temperature was 325 °C and the Orbitrap analyzer scanned over a mass range of *m*/*z* 81–1000 for a full scan at a mass resolution of 70,000. The tandem mass spectra (MS/MS) experiments were performed with a higher collision energy dissociation (HCD) scan. The normalized collision energy was set at 30 eV. Dynamic exclusion was implemented to remove some unnecessary information within the MS/MS spectra [25,26]. (Thermo, QE Plus).

### 2.8. Statistical Analysis

The data were shown as the mean ± standard deviation. Origin 2018 was used for plotting graphs, and IBM SPSS Statistics 25 software was used for data analyses. The Duncan’s test was used for one-way analysis of variance analyses (ANOVA) between groups, and differences were considered statistically significant if *p* < 0.05 (using a 95% confidence limit).

## 3. Results

### 3.1. Effects of Serum Parameters and Cecal Tissue Parameters

Insulin is the only substance in the body that causes hypoglycemia, and its secretion state and receptor function is associated with diabetes [27]. The INS in the MG was significantly reduced than that in the BG, indicating the destruction of pancreatic islet cells. Moreover, their secretory function was inhibited after successfully establishing the model. GHb and GSP levels were directly proportional to the blood glucose concentration. GHb and GSP of the MG group were significantly higher than those of the BG group (*p* < 0.05). The GHb and GSP of the DG group were significantly lower than those of the MG group, indicating that HAS intake can promote insulin secretion (Table 1). Compared to the BG group, four serum lipid parameters in the MG group were significantly altered. TC, TG, LDL-C were significantly increased, and HDL-C was significantly decreased (*p* < 0.05). HAS intervention decreased serum TC and LDL-C and significantly decreased TG. However, HDL-C was significantly increased. Compared to the BG group, the total mass and surface area of the cecum within the MG group were significantly enhanced. However, the mass of the cecal wall had an increasing trend but was not significant (Table 1). 

Compared to the MG group, the cecal wall mass and surface area in the DG group were significantly reduced, and the total mass of the cecum tended to decrease insignificantly. It was shown that HAS could improve cecal tissue enlargement in insulin-resistant mice induced by a high-fat and high-sugar diet (Table 2).

### 3.2. 16S rRNA Gene Amplicon Sequencing

#### 3.2.1. Diversity Analysis

**Figure 1:**
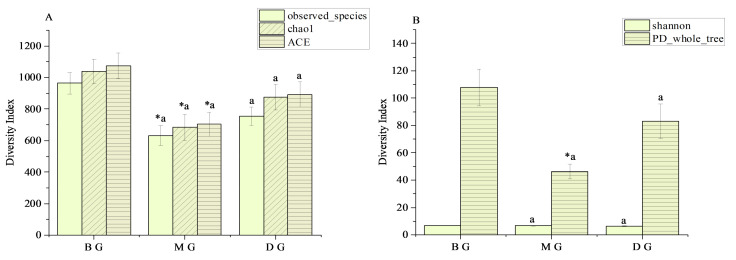
Alpha Diversity index statistics. (**A**) Species richness index; (**B**) Species diversity index. * indicates significant difference between the means of the MG group and the BG group (*p* < 0.05), and the different lowercase superscript letters indicate that the means of the MG group and the DG group are statistically significant (*p* < 0.05). Data were presented as mean ± standard deviation, calculated from three replicates.

The species diversity of gut microbiota in mice during each treatment group is demonstrated in Figure 1. Compared to the BG group, the ACE index, Chao1 index, and Observation-species index of the MG group were significantly reduced (*p* < 0.05) (Figure 1A), and the PD-whole tree index was also significantly reduced (*p* < 0.05) (Figure 1B). Compared to the MG group, the ACE index, Chao1 index, and Observed-species index of the DG group depicted an upward trend (Figure 1A). The results revealed that the MG group decreased the species diversity and richness of the gut microbiota. In contrast, the DG group tended to recover from the decline in the species diversity and richness of the intestinal microbiota of mice, but this was not evident (Figure 1).

#### 3.2.2. Relative Abundance of Gut Microbiota

**Figure 2:**
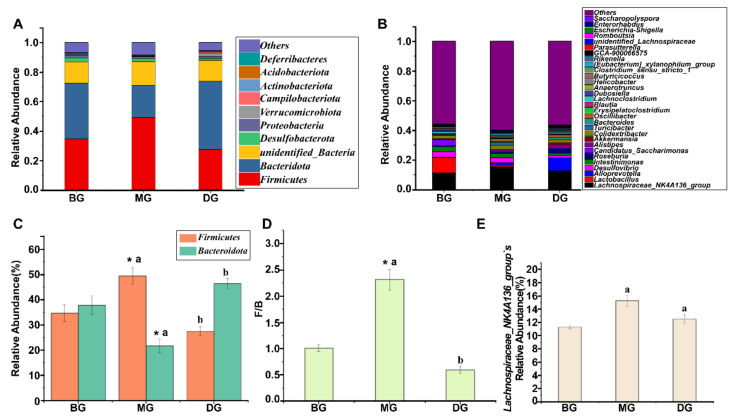
Phylum and genus-level relative abundance. (**A**,**C**,**D**) Phylum horizontal relative abundance; (**B**,**E**) Genus horizontal relative abundance. * indicates significant difference between the means of the MG group and the BG group (*p* < 0.05), and the different lowercase superscript letters indicate that the means of the MG group and the DG group are statistically significant (*p* < 0.05). Data are presented as mean ± standard deviation, calculated from three replicates.

At the phylum level, the gut microbiota composition of mice in each group was the same. The figure shows that the top two dominant phyla based on relative abundance are *Firmicutes* and *Bacteroidota* (Figure 2A). Compared to the BG group, the *Firmicutes* and *Firmicutes/Bacteroidota* (F/B) values of the MG group were significantly elevated, while the *Bacteroidetes* were significantly reduced. Compared to the MG group, the *Firmicutes* and F/B values of the DG group were significantly reduced, while the *Bacteroidota* were significantly elevated (Figure 2C,D). The results showed that the intestinal microbiota of the mice in the MG group was disturbed, and the intestinal microbiota in the DG group had an evident recovery trend.

*Lachnospiraceae_NK4A136*_group was dominant at the genus level in the intestinal microbiota composition in each group (Figure 2B). Compared to the BG group, the relative abundance of *Lachnospiraceae_NK4A136*_group was elevated in the MG group. In contrast, the DG group decreased the relative abundance of *Lachnospiraceae_NK4A13*6_group compared to the MG group (Figure 2E).

### 3.3. Metabonomics Analysis

#### 3.3.1. PCA and OPLS-DA Analysis

**Figure 3:**
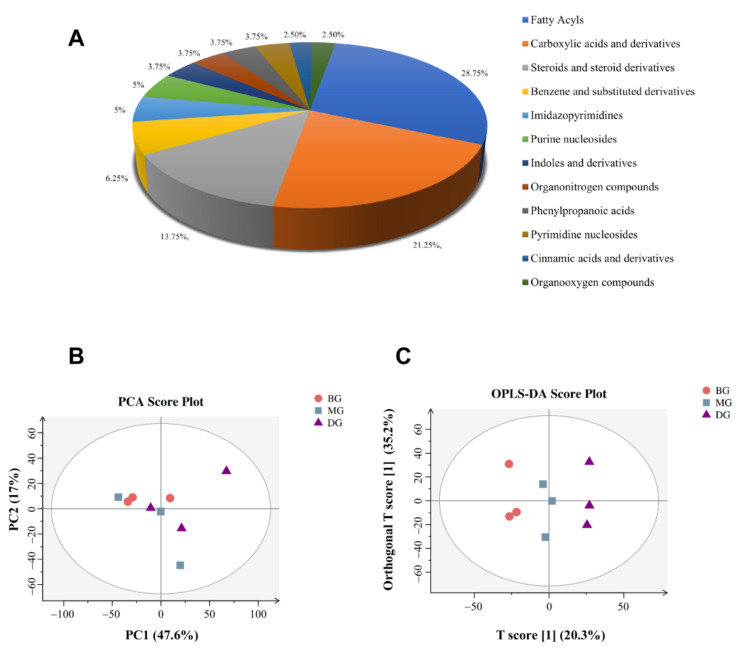
(**A**) Classification map of metabolites; (**B**,**C**) PCA and OPLS-DA charts.

In this experiment, the data were subjected to autoscaling, mean-centering, and scaled to unit variance (UV) processing before multivariate statistical analysis. Principal Component Analysis (PCA) generated new characteristic variables by a linear combination of metabolite variables according to a certain weight, classifies each group of data through main new variables (principal components), and removes samples with poor repeatability (outlier samples) and abnormal samples (in the confidence interval-samples outside the Hotelling T2 ellipse). PCA and Orthogonal Projections to Latent Structures Discriminant Analysis (OPLS-DA) analyses were performed to determine the overall metabolomics differences within the cecal content samples from each group. PCA analysis was performed on the metabolites and the PCA chart showed that PC1 = 47.6%, PC2 = 17%, R^2^ = 0.65, which was greater than 0.5, indicating that the goodness of fit was good, and the model had high explanatory power. In order to further determine the differential metabolites in the cecal contents of the mice in each group, the important differential metabolites between the mice in each group were screened and identified by using multivariate OPLS-DA and univariate *t*-test analysis (Figure 3B,C). In general, variable importance for the projection (VIP) value can be used to illustrate the importance of the variable to explain the X data set and the associated Y data set. When the VIP > 1, it meant that the variable was important, which was regarded as one of the screening criteria for potential biomarkers.

The metabolites in the samples were identified by UPLC/HRMS global untargeted metabolomics technology to clearly understand the rule of metabolite changes in the cecal contents of mice in each group. A total of 101 metabolites were identified (Table S1). Firstly, metabolites procured after identifying all the measured molecular weights were classified according to KEGG and Metabolon.inc. Secondly, the TOP10 categories were selected to draw the pie chart (Figure 3A).

#### 3.3.2. Differential Metabolite Analysis

**Figure 4:**
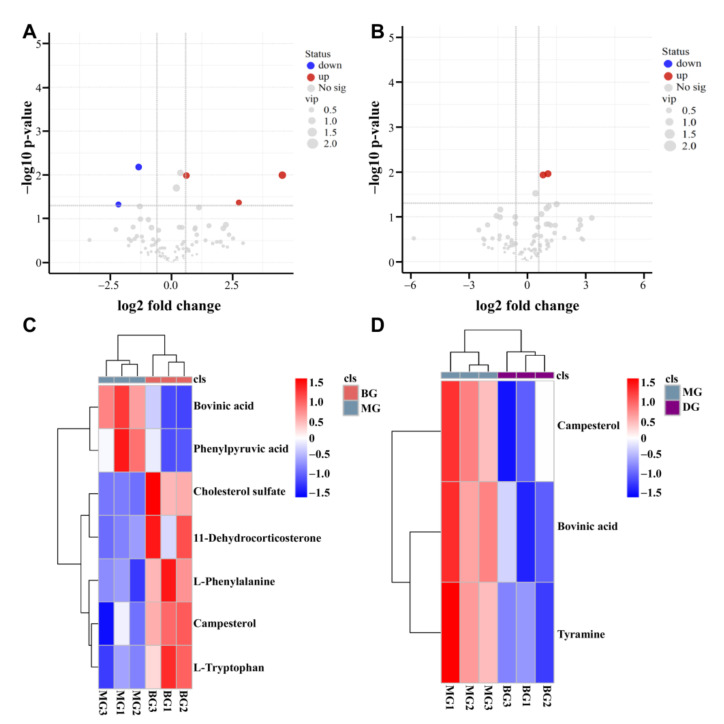
Statistical map of differential metabolites. (**A**,**C**) Map of differential metabolites between BG and MG groups; (**B**,**D**) Map of differential metabolites between MG and DG groups.

From the above OPLS-DA model, metabolites were identified with the screening conditions of VIP > 1 and *p* < 0.05. The metabolites with significant differences were selected based on the Fold-Change ≥ 1.5 or Fold-Change ≤ 0.667 and *p* < 0.05. The metabolites differentially expressed in the two groups were visualized through a volcano plot (Figure 4A,B). The results showed that there were seven differential metabolites in the comparison between the BG group and the MG group, of which three were significantly upregulated, and two were significantly downregulated. In the comparison between the MG group and the DG group, there were three different metabolites, of which two were significantly upregulated (Figure 4C,D).

Based on the basic information from these differential metabolites, we identified that the metabolites were mainly lipids and amino acids (Table 3). The different metabolites from different comparison groups were associated with the KEGG database to obtain the pathway information of the included metabolites. Enrichment analysis was conducted on the annotated results to secure the pathway with more enriched differential metabolites.

**Figure 5:**
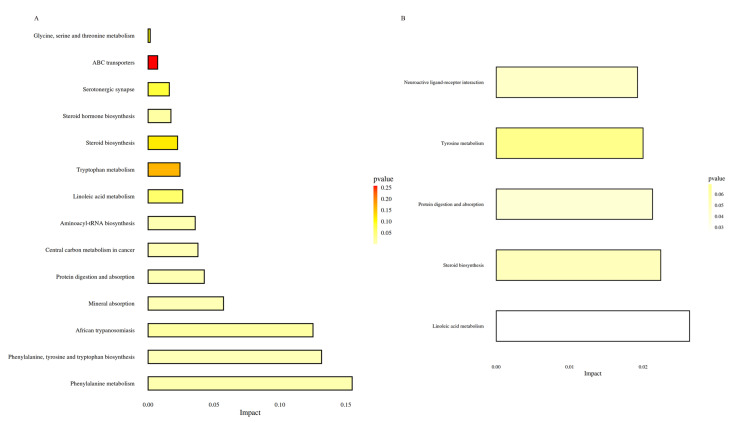
Histogram of metabolic pathway impact factors. (**A**) Histogram of metabolic pathway in BG vs. MG groups; (**B**) Histogram of metabolic pathways in MG vs. DG groups.

The differential metabolites from the BG and MG groups were primarily annotated and enriched in the ABC transporter pathway, African trypanosomiasis pathway, Aminoacyl-tRNA biosynthesis pathway, central carbon metabolism in cancer pathway, glycine, serine and threonine metabolism pathway, linoleic acid metabolism pathway, mineral absorption pathway, phenylalanine metabolism pathway, phenylalanine, tyrosine, and tryptophan biosynthesis pathway, protein digestion and absorption pathway, tryptophan metabolism pathway, steroid hormone pathway, steroid biosynthesis pathway, and serotonergic synapse pathway (Figure 5A). On the other hand, the differential metabolites between the MG and DG groups were mainly associated and enriched in the linoleic acid metabolism pathway, steroid biosynthesis pathway, protein digestion and absorption pathway, tyrosine metabolism pathway, and neuroactive ligand-receptor interaction pathway (Figure 5B).

### 3.4. HE Staining

**Figure 6:**
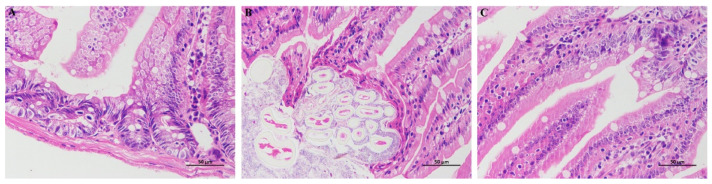
Section analysis of small intestine. (**A**) Small intestine section of BG group; (**B**) Small intestine section of MG group; (**C**) Small intestine section of DG group.

The results of HE showed that the intestinal villi in BG were abundant and structurally complete. There were abundant lamina propria intestinal glands with more goblet cells and no evident inflammation (Figure 6A). Compared with BG, a small number of epithelial cells necrosis and abscission, a small amount of muscle fiber necrosis and enhanced eosinophilic cytoplasm were observed in the mucosal layer of small intestine tissue of MG (Figure 6B); Compared with MG, the intestinal villi in DG were rich in number and complete in structure, a small amount of epithelial degeneration was seen in the mucosal layer, and the morphological structure of muscle fibers was normal and arranged regularly (Figure 6C). The results showed that HAS intervention could improve the atrophy and abscission of the intestinal villi in IR mice.

## 4. Discussion

Diabetes mellitus is a glucose, lipid, protein, and water and electrolyte metabolism syndrome caused by IR or insufficient insulin secretion [28]. STZ selectively destroys pancreatic beta cells, leading to inadequate insulin secretion and diabetes. Therefore, STZ is generally used to develop diabetes models [29]. Our study observed that the INS of insulin-resistant mice was significantly increased when treated with HAS for 28 days, and the GSP and GHb were significantly reduced. Furthermore, the intervention of HAS significantly decreased the swelling of cecal tissue (Table 1). 

Gut microbiota shapes neurological and mental health states, and any type of imbalance in gut microbiota or metabolite production may be associated with imbalances at the central nervous system level, leading to disease. Animal studies show that gut microbiota and its genome influence alterations in energy balance and immunity, leading to metabolic dysfunction [30,31]. The intestinal microbiota plays a vital role in the acquisition and regulation of nutrition and is a crucial factor in the occurrence and development of metabolic disorders [32]. Several studies have established that intestinal microbiota disorder may be an important reason for the pathogenesis of T2DM. Studies have also shown that the changes in the ratio of Firmicutes and Bacteroidetes may accelerate the energy intake of the body and cause obesity [33]. Some studies have used 16S rRNA gene amplicon sequencing, metagenomic sequencing, and metabolomics to analyze the composition and function of intestinal microbiota. Some research results showed that the diversity of intestinal microbiota in Goto–Kakizaki (GK) rats was reduced, and the distribution of microbiota and the interaction between communities changed. In addition, the relationship was altered, which established that the intestinal microbiota was closely related to the T2DM pathogenesis [34].

Observed-species index, chao1 index, and ACE index all represent the richness of the species sample, while Shannon and PD-whole tree index represent the diversity of the species sample [35]. Firmicutes digest nutrients in the gut and maintain host health. The abundance of Bacteroidetes in the gut is closely related to the occurrence and development of obesity [36]. Studies have identified that HFD significantly reduces the relative abundance of Bacteroidetes and elevates the F/B ratio at the phylum level, leading to intestinal dysbiosis [37], which was consistent with our results. This experimental study depicted decreased alpha diversity and altered gut microbiota composition in MG mice based on 16S rRNA gene amplicon sequencing results. Compared to BG mice, a recovery trend was observed in alpha diversity in DG mice.

In the present study, UPLC/HRMS technology combined with metabonomics was used to explore the preliminary effect of HAS on intestinal metabolism within insulin-resistant mice. The BG and MG groups identified seven different metabolites, including L-phenylalanine, L-tryptophan, and Campesterol, involving 14 metabolic pathways. The pathways included phenylalanine metabolism, mineral absorption, protein digestion, and digestion. In addition, three differential metabolites, viz., Bovinic acid, Campesterol, and Tyramine were identified between the MG and DG groups, with five metabolic pathways, including linoleic acid tyrosine metabolism.

There was a positive correlation between increased branched-chain amino acids (BCAAs: valine, leucine, and isoleucine) and IR among obese or diabetic patients. An essential metabolic change associated with IR is amino acid metabolism, particularly BCAAs and aromatic amino acids (AAAs) [38]. L-phenylalanine (L-PHE), L-Tyrosine (L-Tyr), and L-tryptophan (L-TRP) are AAAs [39]. The two kinds of AAAs, tyrosine, and phenylalanine, are associated with diabetes risk [40,41]. Phenylalanine can synthesize essential neurotransmitters and hormones in the human body and participate in the glucose and lipid metabolism of the body. A study involving metabonomic analysis of 251 nonalcoholic fatty liver disease (NAFLD) patients revealed that phenylalanine metabolism disorders were associated with IR [42]. Some studies have established that the tyrosine metabolic pathway is closely associated with IR and has varying degrees of impact on fasting and postprandial blood glucose [43]. In this study, three metabolites of different amino acids were found between the BG group and the MG group. The main metabolic pathways between the BG and the MG groups were Phenylalanine metabolism and Phenylalanine, Tyrosine, and Tryptophan biosynthesis. The differential metabolite of amino acids screened between the MG and DG groups was tyramine, involved in the tyrosine metabolism pathway. Therefore, the metabolism of amino acids could be maladjusted during IR. 

The leading cause of IR is lipid metabolism disturbance [44]. Individuals with T2D typically present with dyslipidemia, with high triglyceride levels, small dense low-density lipoprotein (LDL) particles, low high-density lipoprotein (HDL) cholesterol levels, and normal or near-normal LDL cholesterol levels [45]. In addition, lipid metabolism includes fatty acid metabolism, primary bile acid synthesis, and steroid biosynthesis. The lipid differential metabolites screened in the current study included Cholesterol sulfate, Campesterol, Bovinic acid, and 11-dehydrocorticosterone, and are involved in the biosynthesis of steroids through a common metabolic pathway. Between the BG and MG groups, Cholesterol sulfate and 11-dehydrocorticosterone were significantly upregulated, while Bovinic acid was significantly down-regulated. On the other hand, Bovinic acid was significantly upregulated between the MG and DG groups. The content changes in lipid metabolism-related substances indicated that the body lipid metabolism was a disorder in insulin-resistant mice. The intake of HAS can play a specific regulatory role by improving lipid metabolism.

## 5. Conclusions

Based on 16S rRNA gene amplicon sequencing and metabonomics studies, it was observed that the intestinal diversity, microbiota, lipid metabolism, and amino acid metabolism of insulin-resistant mice were disturbed. The intervention of HAS could improve the intestinal and metabolic state. Therefore, HAS could improve IR symptoms by regulating multiple metabolic pathways. However, the study results require clinical verification. In addition, the specific mechanism of metabolites, lipid metabolism, and amino acid metabolism has not been thoroughly discussed and requires further research.

## Figures and Tables

**Table 1:** Effects of hydroxy-alpha-sanshool on key serum indexes in mice.

	INS (mU/L)	GSP (mmol/L)	GHb (ng/mL)	TC (mmol/L)	TG (mmol/L)	HDL-C (mmol/L)	LDL-C (mmol/L)
BG	60.69 ± 5.86	3.21 ± 0.10	8.66 ± 1.08	3.08 ± 0.48	1.18 ± 0.33	3.43 ± 0.19	0.22 ± 0.03
MG	32.24 ± 4.42 *^a^	6.45 ± 0.42 *^a^	13.66 ± 1.05 *^a^	7.06 ± 0.75 *^a^	2.53 ± 0.18 *^a^	0.87 ± 0.24 *^a^	0.58 ± 0.06 *^a^
DG	52.35 ± 4.22 ^b^	4.09 ± 0.17 ^b^	5.04 ± 1.05 ^b^	5.57 ± 0.38 ^a^	1.75 ± 0.24 ^b^	2.87 ± 0.49 ^b^	0.46 ± 0.10 ^a^

* indicates significant difference between the means of the MG group and the BG group (*p* < 0.05), and the different lowercase superscript letters indicate that the means of the MG group and the DG group are statistically significant (*p* < 0.05). Data were presented as mean ± standard deviation, calculated from ten replicates.

**Table 2:** Effect of hydroxy-alpha-sanshool on cecum index in mice.

	Total Cecal Mass (g)	Cecal Wall Mass (g)	Cecal Surface Area (cm^2^)
BG	0.44 ± 0.17	0.14 ± 0.05	0.51 ± 0.01
MG	0.57 ± 0.04 *^a^	0.19 ± 0.04 ^a^	0.74 ± 0.11 *^a^
DG	0.50 ± 0.09 ^a^	0.12 ± 0.01 ^b^	0.57 ± 0.04 ^b^

* indicates significant difference between the means of the MG group and the BG group (*p* < 0.05), and the different lowercase superscript letters indicate that the means of the MG group and the DG group are statistically significant (*p* < 0.05). Data were presented as mean ± standard deviation, calculated from ten replicates.

**Table 3:** Basic information on differential metabolites.

Name	Formula	*m*/*z*	Rt (s)	Exact Mass	Classification
Cholesterol sulfate	C_27_H_46_O_4_S	467.32	716.49	466.31	Lipid
Campesterol	C_28_H_48_O	383.37	805.14	400.37	Lipid
Bovinic acid	C_18_H_32_O_2_	279.23	788.14	280.24	Lipid
L-Phenylalanine	C_9_H_11_NO_2_	164.07	322.73	165.08	Amino acid
L-Tryptophan	C_11_H_12_N_2_O_2_	203.08	371.93	204.09	Amino acid
11-Dehydrocorticosterone	C_21_H_28_O_4_	325.18	810.04	344.20	Lipid
Phenylpyruvic acid	C_9_H_8_O_3_	163.04	398.84	164.05	Amino acid
Tyramine	C_8_H_11_NO	136.08	442.01	137.08	Amino acid

## Data Availability

The data that support the findings of this study are available from the corresponding author upon reasonable request.

## Supplementary Materials

The following supporting information can be downloaded at: https://www.mdpi.com/article/10.3390/foods11142040/s1, Table S1: Metabolites identified.
